# Marriage, Children, and Sex-Based Differences in Physician Hours and Income

**DOI:** 10.1001/jamahealthforum.2023.0136

**Published:** 2023-03-24

**Authors:** Lucy Skinner, Max Yates, David I. Auerbach, Peter I. Buerhaus, Douglas O. Staiger

**Affiliations:** 1 Geisel School of Medicine at Dartmouth, Hanover, New Hampshire; 2 Cambridge University, Cambridge, United Kingdom; 3 Center for Interdisciplinary Health Workforce Studies, Mark & Robyn Jones College of Nursing, Montana State University, Bozeman, Montana; 4 Department of Economics, Dartmouth College, Hanover, New Hampshire; 5 National Bureau of Economic Research, Cambridge, Massachusetts

## Abstract

**Importance:**

A better understanding of the association between family structure and sex gaps in physician earnings and hours worked over the life cycle is needed to advance policies addressing persistent sex disparities.

**Objective:**

To investigate differences in earnings and hours worked for male and female physicians at various ages and family status.

**Design, Setting, and Participants:**

This retrospective, cross-sectional study used data on physicians aged 25 to 64 years responding to the American Community Survey between 2005 and 2019.

**Exposures:**

Earned income and work hours.

**Main Outcomes and Measures:**

Outcomes included annual earned income, usual hours worked per week, and earnings per hour worked. Gaps in earnings and hours by sex were calculated by family status and physician age and, in some analyses, adjusted for demographic characteristics and year of survey. Data analyses were conducted between 2019 and 2022.

**Results:**

The sample included 95 435 physicians (35.8% female, 64.2% male, 19.8% Asian, 4.8% Black, 5.9% Hispanic, 67.3% White, and 2.2% other race or ethnicity) with a mean (SD) age of 44.4 (10.4) years. Relative to male physicians, female physicians were more likely to be single (18.8% vs 11.2%) and less likely to have children (53.3% vs 58.2%). Male-female earnings gaps grew with age and, when accumulated from age 25 to 64 years, were approximately $1.6 million for single physicians, $2.5 million for married physicians without children, and $3.1 million for physicians with children. Gaps in earnings per hour did not vary by family structure, with male physicians earning between 21.4% and 23.9% more per hour than female physicians. The male-female gap in hours worked was 0.6% for single physicians, 7.0% for married physicians without children, and 17.5% for physicians with children.

**Conclusions and Relevance:**

In this cross-sectional study of US physicians, marriage and children were associated with a greater earnings penalty for female physicians, primarily due to fewer hours worked relative to men. Addressing the barriers that lead to women working fewer hours could contribute to a reduction in the male-female earnings gap while helping to expand the effective physician workforce.

## Key Points

**Question:**

Is family structure associated with the sex gap in physician earnings and hours worked?

**Findings:**

In this cross-sectional study of 95 435 US physicians, the male-female gap in hours worked was 1% for single physicians, 7% for married physicians without children, and 18% for physicians with children. Male physicians earned 21% to 24% more per hour than female physicians across all family structures.

**Meaning:**

This study found that marriage and children were associated with a greater earnings penalty for female physicians primarily because of fewer hours worked; addressing this barrier may help to expand the effective physician workforce.

## Teaser

This cross-sectional study uses American Community Survey data to evaluate differences by sex in physician earnings and hours worked by age and family structure.

## Introduction

It is well established that male physicians earn significantly more than female physicians.^1,2,3,4,5,6,7^ A recent analysis estimated that when accumulated over a 40-year career, female physicians earn approximately $2 million less than male physicians.^8^ The underlying cause of the earnings gap is less clear, however. Some studies have found that the earnings gap is partly explained by practice characteristics and work patterns associated with female physicians working in lower-paying specialties, working fewer hours than male physicians,^4,9^ and generating less practice revenue because they spend relatively more time per patient visit and, therefore, bill less than their male counterparts.^10^

However, the choice of specialty, practice characteristics, billing practices, work hours, and other factors associated with the disparity in physician earnings are mediated, in part, by structural sexism and gender biases.^11^ Structural sexism within the labor market refers to the gendered organization systems and discriminatory practices that lead men to positions of higher compensation compared with women. In addition, structural sexism affects the allocation of roles, responsibility, and authority in family life that systematically disadvantage women in their ability to participate in the labor force.^11^ These factors and others that contribute to the association between physician sex and earnings are depicted in the conceptual framework illustrated in the eFigure in Supplement 1.

Because structural sexism is difficult to observe directly, researchers in other professions have studied the association between family structure and earnings and hours worked for men and women. As women have entered careers in previously male-dominated occupations, the historical responsibility that has been disproportionately placed on women to manage the household and care for children has further contributed to sex gaps in earnings. Studies of business and law school graduates have found that while there were no earnings differences immediately after graduation, a sex gap appeared and grew once graduates had children.^12,13^ In medicine, a study of hours worked among dual-physician couples reported that having children was associated with reduced work hours among female physicians but not among male physicians.^14^ Female physicians also reported differences in time commitments in parenting and performing household tasks,^15^ which for many women is associated with acquiring less experience and job-related skills and a subsequent loss in their human capital relative to men.^16,17,18^ However, there is no recent evidence on how marriage and children are associated with hours and earnings among all physicians, and the relative contribution of differences in hours worked vs differences in earnings per hour to the physician earnings gap has not previously been quantified.

More than one-half of medical students are women, and large numbers of male physicians will retire over the next decade; therefore, health care delivery systems will increasingly rely on female physicians to deliver medical care.^19^ Understanding how family structure is associated with both differences in hours worked between female and male physicians and related sex-based earnings gaps is critical to prepare for an increasingly female physician workforce.

We used national survey data over a 15-year period to investigate differences in earnings and hours worked for male and female physicians at various ages during their careers. We stratified physicians based on whether they were single, married, or had children and analyzed the total male-female earnings gap, the gap in earnings per hour, and differences in hours worked across these groups. We also estimated the accumulated differences in male-female earnings over a 40-year career.

## Methods

Data for this cross-sectional study came from the American Community Survey (ACS),^20^ administered by the US Census Bureau and surveying 1% of the US population each year between 2005 and 2019 (data collection in 2020 was affected by the pandemic and, therefore, not included).^21^ Each respondent reported demographic information, including age, sex, race and ethnicity, rural residence, marital status, and number of their own children living in the household, along with employment information, including occupation, industry, annual earnings, weeks worked per year, and hours worked per week. Physicians were associated with a hospital referral region (HRR) by mapping the public-use microarea of their residence available in the ACS to HRRs using MABLE/Geocorr, version 14 (University of Missouri). Between 9000 and 12 000 individuals reported their occupation as physician or surgeon each year. The ACS is an annual cross-sectional survey and does not include repeated observations on individual physicians. While the ACS provides detailed information on employment, earnings, demographic details, and family structure for a large representative sample of physicians, it does not include information on physician specialty.

Our sample was restricted to physicians who were working in the year of the survey, were between the ages of 25 and 64 years, reported their occupation as physician or surgeon, worked at least 20 hours per week and at least 48 weeks in the previous year, and had annual earnings of at least $10 000. Although many physicians are employed beyond age 64 years and few are younger than 30 years, we focused our analysis on age ranges where physicians might have children at home. Weeks worked included paid leave and when the physician worked only a few hours, so only 8.3% of all physicians reported working less than 48 weeks. Physicians with missing or imputed data for occupation, income, hours, weeks worked, age, sex, and marital status were excluded. The final sample included 95 435 physicians across all years studied.

The sample of physicians was stratified into 3 categories to assess the association of marriage and children with the earnings gap: single without children (n = 11 725), married without children (n = 29 030), and with children (n = 54 680). A physician was classified as single if they reported never being married or as widowed, divorced, or separated. A physician was classified as with children if they had 1 or more children living in their household, regardless of marital status. Because the ACS does not identify children not living in the household, older physicians whose children have left the household were classified as without children.

Hours worked were measured as the respondent’s reported usual weekly hours worked. Hourly wages were calculated as annual earnings divided by usual weekly hours times 50 weeks.

Earnings for each physician were calculated as the sum of self-reported wage and salary income plus business income during the previous year. For privacy reasons, income for the top 1% of earners in each state was recoded by the US Census Bureau as the average value for all individuals in the top 1% of earnings. This adjustment has been found to have little effect on estimates of physician earnings.^21^ Because comparisons of ACS estimates of income with estimates from tax records suggest that physicians may underreport business income in the ACS, our estimates do not account for sex disparities in unreported business income.^21^ This study was deemed exempt from institutional review board or ethics committee review because the data used were from a public-use government-administered survey. The study followed the Strengthening of Reporting Observational Studies in Epidemiology (STROBE) reporting guideline.

### Statistical Analysis

Statistical analyses were conducted between 2019 and 2022. The average earnings and other physician characteristics were calculated by sex and age group overall and separately for physicians who were single, married, and with children. We adjusted observed earnings gaps between men and women using multiple regression to control for the following physician characteristics: race (9 categories), ethnicity (5 categories), citizenship status (4 categories), rural residence (2 categories), HRR of residence (306 categories), spouse’s education (6 categories), single year of age, and indicators for the year of the survey. These are external factors that could be associated with earnings. However, adjusted and unadjusted estimates of earnings gaps were similar (eTable 1 in Supplement 1). On the other hand, we did not control for specialty, practice setting (hospital, private practice, etc), or other characteristics that could be part of conceptual framework for the association between sex and earnings (eFigure in Supplement 1). To deconstruct the percent difference in earnings between male and female physicians of each family type into differences in earnings per hour and differences in hours worked, we performed regression analyses of the log of earnings, hours, and earnings per hour on a male-female indicator, controlling for physician characteristics. Lifetime earnings differences were calculated by summing the earnings gaps at each age between age 25 and 64 years based on regression coefficients. While this calculation is not based on longitudinal estimates for a cohort of individuals, it is a useful summary measure of differences across groups (analogous to the use of total fertility rates).

To be nationally representative, all analyses were weighted by sampling weights provided by the ACS. We report SEs that account for the use of sampling weights and for clustering within households. We used *t* tests to test differences in earnings per hour and hours worked, with a 2-sided *P* < .05 considered significant. Statistical analysis was performed using Stata, version 16.0 software (StataCorp LLC).

## Results

Characteristics of our sample of male and female physicians between 2005 and 2019 are shown in the Table. The sample included 95 435 physicians (35.8% female and 64.2% male; 19.8% Asian, 4.8% Black, 5.9% Hispanic, 67.3% White, and 2.2% other race or ethnicity; mean [SD] age, 44.4 [10.4] years). Female physicians were younger than male physicians, with a mean (SD) age of 41.9 (9.9) years vs 45.8 (10.5) years, respectively, and female physicians were more likely to identify as a minoritized race or ethnicity, with 37.8% of women (vs 29.8% of men) identifying as Asian, Black, Hispanic, or other (non-White). Adjusted to 2019 dollars, average earnings among male physicians were $91 000 higher than among female physicians. Male physicians also worked 4.4 more hours per week and were more likely to be self-employed than female physicians (24.9% vs 14.6%). Regarding family structure, female physicians were more likely than male physicians to be single (18.8% vs 11.2%) and less likely to have children (53.3% vs 58.2%). Among those who were married, female physicians were less likely to report their spouse as not working (11.0% vs 41.6%). The spouses of female physicians earned 3 times as much as the spouses of male physicians ($137 000 vs $51 000) and were more likely to have a professional or doctoral degree (46.1% vs 29.1%). Sex differences in race and ethnicity and spousal characteristics were more pronounced among older physicians (eTable 2 in Supplement 1), which may have contributed to larger earnings gaps at older ages.

**Table.** Characteristics of Male and Female Physicians, 2005-2019

	Physicians, %	
	Female (n = 34 204)	Male (n = 61 231)
Demographic characteristics		
Age, mean (SD), y	41.9 (10.5)	45.8 (9.9)
Race and ethnicity		
Asian	22.2	18.3
Black	6.8	3.6
Hispanic	6.1	5.9
White	62.2	70.2
Other^a^	2.7	1.9
Employment		
Average earnings, 2019 $	194 000	285 000
Usual hours worked per wk	50.9	55.3
Self-employed	14.6	24.9
Hospital	48.9	42.8
Physician office	31.4	39.3
Family status		
Single, no children	18.8	11.2
Married, no children	27.9	30.5
With children	53.3	58.2
Spousal characteristics^b^		
Not working	11.0	41.6
Average earnings, $	137 000 (144 000)	51 000 (88 000)
Professional or doctoral degree	46.1	29.1
Physician, lawyer, or manager	46.1	23.9

^a^ Other includes American Indian or Alaska Native, non-Hispanic, other race or ethnicity not elsewhere classified, 2 major races or ethnicities, and 3 or more major races or ethnicities.

^b^ Averages of spousal characteristics are for 51 411 male and 24 774 female physicians who were married and had a spouse reporting data.

**Figure 1.**
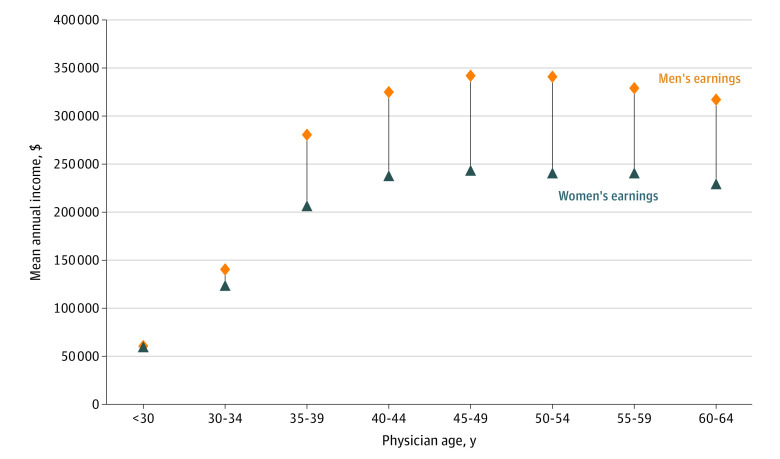
Annual Earnings for Male and Female Physicians by Age Group Data represent 95 435 physicians observed between 2005 and 2019. Physicians working fewer than 20 hours per week and aged younger than 25 years or older than 64 years are excluded.

Average annual earnings for male and female physicians by 5-year age groupings are shown in Figure 1. The difference was negligible for physicians younger than 30 years, relatively small for physicians aged 30 to 34 years ($16 733, or 12% higher for male physicians), but then grew to $74 345 (26.5% higher for male physicians) at age 35 to 39 years at a time when child-rearing is common. The earnings gap peaked at $100 129 (29.4% higher for male physicians) at age 50 to 54 years before declining somewhat for older physicians.

**Figure 2.**
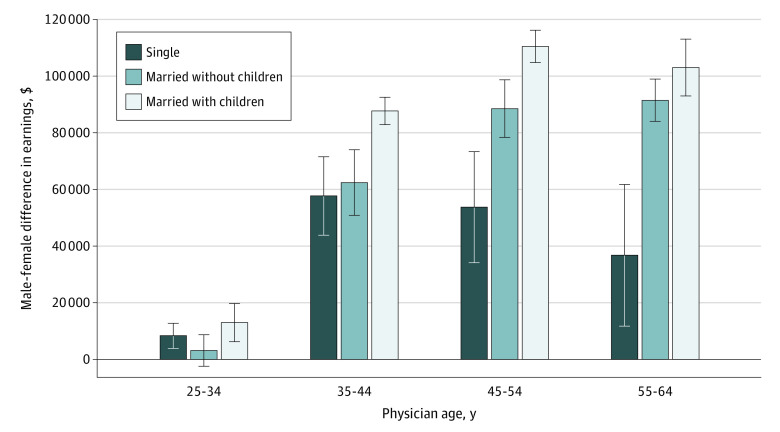
Male-Female Earnings Gap by Physician Marital Status and Age Data are shown as SEs based on 95% CIs. Earnings gaps are adjusted for physician race and ethnicity, citizenship, hospital referral region, rural residence, and spousal education. Single physicians have never been married, while married physicians include those previously married as well as currently married. Married with children includes any respondent with children in their household.

Figure 2 shows the male-female earnings gap by physician age and family structure, adjusted for physician characteristics. The large earnings gaps starting in the mid-30s were not uniform across family structure. The gaps were largest for physicians who were married with children, peaking at $110 000 in the 45- to 54-year age range. While the gap is also relatively large for both single and married physicians without children, these 2 groups diverged as they approached older ages, with the gap for single physicians decreasing (ie, male-female earnings for single physicians are closer together among older physicians) and the gap for married physicians growing toward that for physicians with children.

**Figure 3.**
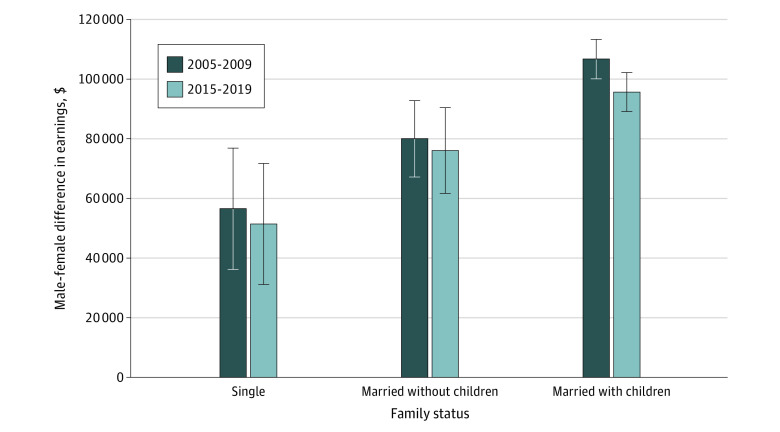
Earnings Gap for Physicians Aged 35 to 54 Years in 2005-2009 and 2015-2019 by Family Status Data are shown as SEs based on 95% CIs. Earnings gaps are adjusted for physician race and ethnicity, citizenship, hospital referral region, rural residence, and spousal education. Single physicians have never been married, while married physicians include those previously married and currently married. With children includes any of a respondent’s own children in their household. Sample sizes are 17 650 (2005-2009) and 18 324 (2015-2019).

To evaluate whether the earnings gap changed over time, we focused on physicians aged 35 to 54 years, where the gap is the largest. Figure 3 shows the comparison of the mean earnings gap among the different family structures during the first 5 years of our data (2005-2009) to the last 5 years (2015-2019). While the earnings gap declined slightly for all groups of physicians aged 35 to 54 years, the size of the gap remained large. From 2005 to 2009, single male physicians earned $57 000 more than single female physicians, while from 2015 to 2019, this gap decreased to $51 000. Similarly, over this same period, the gap between male and female earnings for physicians with children decreased by approximately $11 000, from $107 000 to $96 000. While these changes were not jointly statistically significant (*P* = .13), they suggest a small shrinkage in the earnings gaps across the 10-year period.

**Figure 4.**
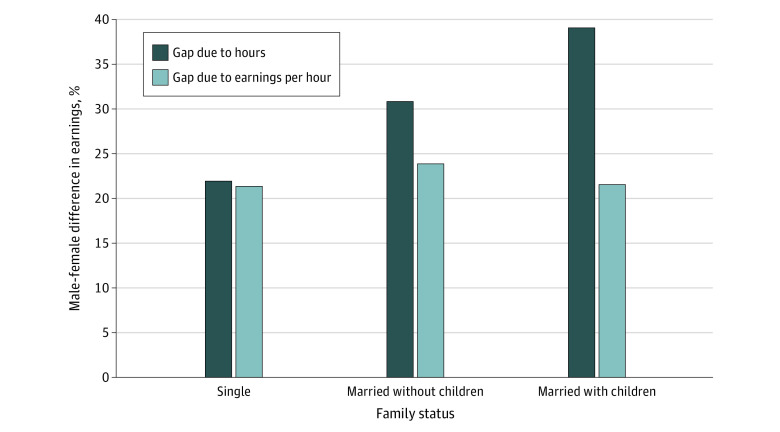
Earnings Gap Between Male and Female Physicians Aged 35 to 54 Years by Portion of the Gap Due to Hours Worked vs Earnings per Hour Bars represent the percent difference in earnings between male and female physicians of each family type due to differences in earnings per hour and total hours worked separately. Data are based on coefficients from a regression of the log of earnings, including adjustment for the same factors in earlier analyses.

Figure 4 deconstructs the male-female earnings gap (measured as the percent difference in earnings) into the portion due to differences in earnings per hour and the portion due to differences in hours worked, on average (also focusing on physicians aged 35-54 years). The gap in earnings per hour between male and female physicians was similar for all family structures (single physicians, 21.4% [95% CI, 16.0%-26.7%]; married physicians, 23.9% [95% CI, 20.0%-27.7%]; physicians with children, 21.5% [95% CI, 19.9%-23.2%]). In contrast, the gap in hours worked between male and female physicians differed substantially across family types, with marriage and the presence of children at home associated with a disproportionate reduction in hours worked by female physicians. Single male and female physicians work approximately the same number of hours per week (difference, 0.6%; 95% CI, −1.5% to 2.7%), while married female physicians worked 7.0% (95% CI, 5.6%-8.4%) fewer hours than married male physicians, and female physicians with children worked 17.5% (95% CI, 16.8%-18.2%) fewer hours than male physicians with children. Thus, the greater male-female earnings gap among physicians who were married and those with children was largely due to a greater male-female gap in hours worked rather than differences in earnings per hour.

When accumulated over a lifetime (assuming a 40-year career as a physician), the difference in earnings is large. The lifetime male-female earnings difference is approximately $1.6 million for physicians who remained single over their lifetimes, $2.5 million for physicians who were married without children, and $3.1 million for physicians with children. Because most physicians spent some of their careers in more than 1 of these family types and have careers shorter than 40 years, actual earnings differences may be less than these estimates.

## Discussion

Using nationally representative data on more than 95 000 physicians between 2005 and 2019, we found that female physicians earn less than male physicians for all ages and family types. Marriage and children were associated with an additional earnings penalty for female physicians, which is primarily due to fewer hours worked relative to male physicians. Among physicians with children, the male-female gap in earnings over a 40-year career exceeded $3 million, more than double the gap for single physicians. These gaps have been persistent, changing little over the past 15 years.

Our findings align with a well-established body of evidence showing that there is a significant gap between female and male physician earnings that has persisted over time. The large increase in the female-male earnings gap over the life cycle is surprising in such a highly skilled profession where both men and women have invested substantially in their career. However, Bertrand et al^12^ found similar outcomes for women with an MBA degree, where having a child during their career was associated with less accumulated job experience, more career disruptions, shorter work hours, and a significant decline in earnings compared with men with an MBA degree with children.

We found that marriage and children are associated with a greater earnings penalty for female physicians primarily due to fewer hours worked relative to men. Among single physicians, there was no difference in hours worked between men and women. However, among married physicians, women worked 7% fewer hours than men, and among physicians with children, women worked 18% fewer hours than men. These gaps in hours worked are similar to those found in a previous study using a smaller sample of physicians from the 1990s.^7^ This differential could be associated with an uneven division of the burden of household and parenting tasks within the couple, as has been found in earlier studies of physicians in the US and Canada.^7,22^ One potential reason why male physicians work more hours and shoulder a lesser burden at home may be that married male physicians may have spouses who are not working, are lower paid, and are less highly educated compared with spouses of female physicians (Table). Nevertheless, the female-male gap in hours worked persisted, even when controlling for spousal education, suggesting that other factors may be at work.

In contrast to the hours gap, our findings indicate that the gap in earnings per hour is not strongly associated with marriage and children, where female physicians earned between 21.4% and 23.9% less per hour than male physicians, regardless of whether they were single, married, or had children. This difference in earnings per hour may be associated with women choosing to practice in lower-paying specialties.^23,24^ Such decisions have been attributed to unconscious beliefs and overt sexist attitudes and behaviors during undergraduate and graduate medical education^22,24^: benevolent sexism, where medical school advisers and mentors encourage women to enter more empathetic specialties; and hostile sexism, where medical students experience overt antipathy during their experiences with certain specialties on the basis of their sex.^25,26^ Additionally, the greater willingness of male physicians to negotiate for higher salaries and the preferential choice of male physicians for positions of leadership could further contribute to differences in earnings.^17,18,23^

Our analysis does not control for factors associated with structural sexism within the conceptual framework between physician sex and earnings (eFigure in Supplement 1). In studies using race and ethnicity as exposure variables in analyses of health outcomes, there is an argument to forgo controlling for variables such as neighborhood and family socioeconomic status, as these variables are within the causal order.^27,28^ Similarly, we chose not to control for specialty choice and academic and leadership positions because these factors are mediated by structural sexism.^6,25^ Female physicians are not inherently less able to become surgeons or to achieve academic and leadership positions; rather, the internalized sexism, cultural biases, assumptions of allocation of family duty, and stigmas about working women may have contributed to specialty choice and the preferential treatment of male physicians with regard to jobs, earnings, and promotion.

The suggestion by Gottlieb and Jagsi^18^ that health care delivery organizations use quality improvement programs to address inequities in physician earnings could also be applied to the difference in hours worked by sex. Physicians are increasingly becoming employees of hospital systems, which gives these large-scale employers the opportunity to better support women in the workforce and to give female physicians greater freedom to choose historically male-dominated specialties and receive pay commensurate with hours worked.^29^ Beyond identifying whether current strategies such as day care and flexible work hours make a positive difference, applying a root cause analysis and other quality improvement techniques could identify whether, and why, female physicians earn less per hour and work fewer hours than male physicians, as well as factors associated with patient care and the organization’s financial health. Of course, not all female physicians prefer to work full time, but some may, and organizations should test strategies that support women in their career goals, such as increasing paid maternity and paternity leave and supporting onsite childcare services.

As the proportion of female physicians increases over the decade,^19^ educators and health care delivery systems must assess the structure of physician work and pay to ensure equity. Brubaker^17^ described the disproportional effects of the COVID-19 pandemic experienced by female physicians and has urged organizations to use the pandemic as a catalyst to address sex-based earnings inequities and improve the life-work balance for all physicians. As national leadership focuses on preparing the future physician workforce, we have reached a critical moment to address sex equity in physician reimbursement within health care institutions. Addressing the barriers that lead to women making less per hour and working fewer hours could achieve the dual aims of reducing male-female earnings disparities while expanding the effective physician workforce.

### Limitations

This study has several limitations. First, comparisons of physician income reported in the ACS with that reported on tax forms suggest that business income is underreported in the ACS.^21^ This underreporting may have resulted in estimates that understate the female-male gap in earnings, particularly as the male physicians in our sample were more likely to be self-employed. Second, the ACS does not include physician specialty or detailed practice information and, therefore, does not allow for an evaluation of patterns by specialty. Our conceptual framework suggests that estimates that do not adjust for specialty include the mediating effects of sex on specialty choice through structural sexism. Nevertheless, previous studies have found that estimates of earnings gaps for physicians by sex persist after adjustment for specialty and characteristics of practice. One study that used a nationally representative sample of physicians found that 70% to 80% of the male-female earnings gap persisted after adjustment for physician specialty and practice information.^30^ Another study of academic physicians found that 40% of the male-female earnings gap persisted after further controlling for faculty rank, specialty, scientific authorship, National Institutes of Health funding, clinical trial participation, and Medicare reimbursements (in addition to specialty).^6^ Third, our analysis relies on self-reported hours worked, which may be underreported for female physicians. Female physicians spend more time on electronic health records outside of business hours and on unscheduled days compared with their male colleagues.^31^ However, independent measures of working time have corroborated self-reported hours in census data, and discrepancies were not associated with respondent characteristics such as age, sex, marital status, or presence of children.^32^ While this underreporting would narrow the gap in hours worked between male and female physicians, it would lead to an increase in the gap in earnings per hour, as female physicians are not compensated for these additional hours of work.

## Conclusions

In this cross-sectional study of US physicians, marriage and children were associated with a greater earnings penalty for female physicians primarily due to fewer hours worked relative to men. Female physicians, regardless of family structure, earn less per hour than male physicians. Addressing the barriers that lead to women working fewer hours and earning less per hour over their career course could contribute to a reduction in the male-female earnings gap while expanding the effective physician workforce.

## Notes

Supplement 1. **eFigure.** Conceptual Framework for the Association Between Physician Sex and Earnings **eTable 1.** Characteristics of Male and Female Physicians by Age Group, 2005-2019 **eTable 2.** Robustness of Earnings Gap Estimates to Alternative Control Variables

Supplement 2.
Data Sharing Statement
