# Supplementary material for: Marriage, Children, and Sex-Based Differences in Physician Hours and Income
Source: JAMA Health Forum. 2023 Mar 24;4(3):e230136. doi: 10.1001/jamahealthforum.2023.0136 (PMC10313148; doi:10.1001/jamahealthforum.2023.0136)
Supplement: Supplement 1. — eFigure. Conceptual Framework for the Association Between Physician Sex and Earnings eTable 1. Characteristics of Male and Female Physicians by Age Group, 2005-2019 eTable 2. Robustness of Earnings Gap Estimates to Alternative Control Variables [file jamahealthforum-e230136-s001.pdf]

Supplemental Online Content

Skinner L, Yates M, Auerbach DI, Buerhaus PI, Staiger DO. Marriage, children, and sex-based differences in physician hours and income. *JAMA Health Forum.* 2023;4(3):e230136. doi:10.1001/jamahealthforum.2023.0136

eFigure. Conceptual Framework for the Association Between Physician Sex and Earnings

eTable 1. Characteristics of Male and Female Physicians by Age Group, 2005-2019

eTable 2. Robustness of Earnings Gap Estimates to Alternative Control Variables

This supplemental material has been provided by the authors to give readers additional information about their work.

© 2023 Skinner L et al. *JAMA Health Forum*.

eFigure. Conceptual Framework for the Association Between Physician Sex and Earnings

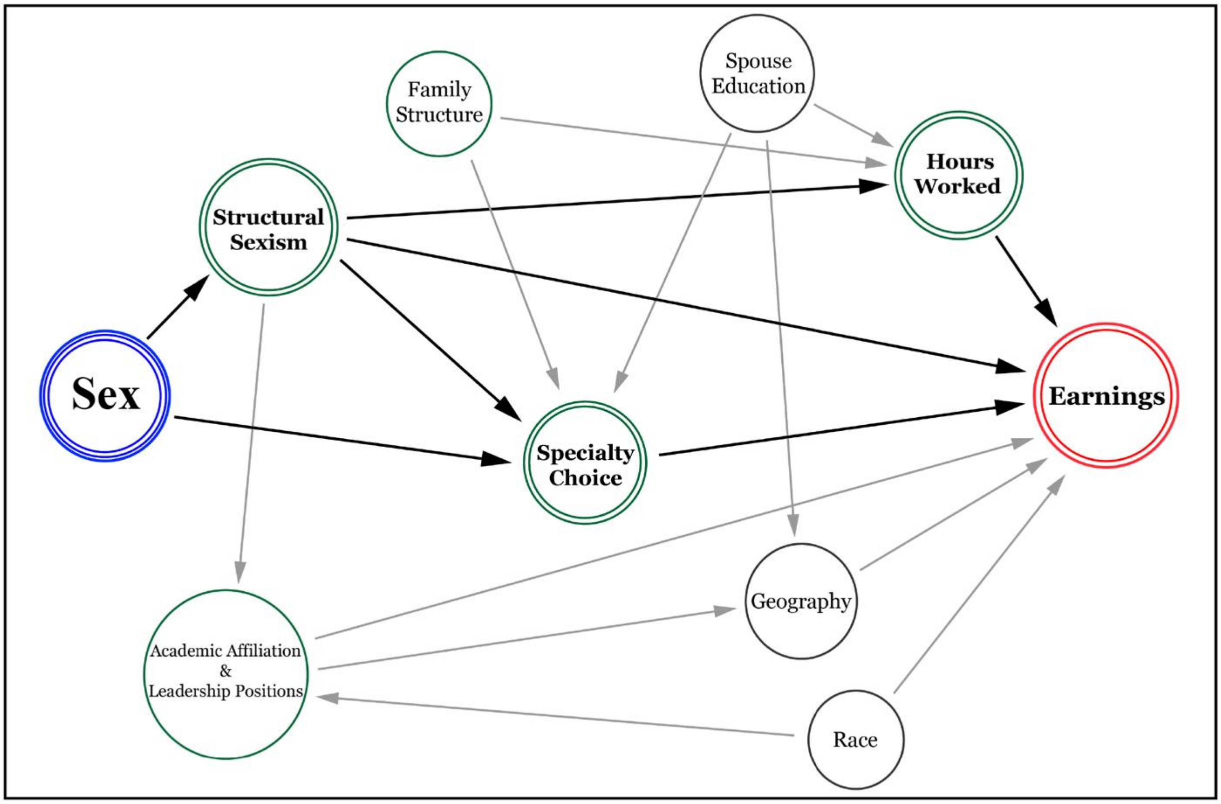

© 2023 Skinner L et al. *JAMA Health Forum*.

eTable 1. Characteristics of Male and Female Physicians by Age Group, 2005-2019

	**Age 25-34**		**Age 35-54**		**Age 55-64**	
	**Male Physicians**	**Female Physicians**	**Male Physicians**	**Female Physicians**	**Male Physicians**	**Female Physicians**
Number	10,254	9,223	33,864	19,697	17,113	5,284
Demographics						
Age (years)	30.9	30.7	44.6	43.5	59.2	58.8
White non-Hispanic	61.8%	59.1%	69.0%	61.8%	78.9%	70.6%
Black non-Hispanic	3.8%	6.3%	3.9%	7.1%	3.1%	6.4%
Asian non-Hispanic	25.5%	24.8%	19.0%	22.3%	11.8%	16.2%
Hispanic	5.8%	6.3%	6.2%	6.2%	5.1%	4.9%
Other	3.0%	3.4%	2.0%	2.5%	1.1%	2.0%
Employment						
Average Earnings (2019 $)	$118,000	$103,000	$322,000	$230,000	$324,000	$236,000
Usual Hours worked per week	61.2	58.6	54.6	48.0	52.6	46.9
Self Employed	3.3%	2.7%	26.6%	17.4%	36.8%	27.9%
Works in Hospital	72.2%	70.8%	38.9%	41.5%	30.3%	33.6%
Works in Physician Office	12.4%	13.2%	42.5%	37.8%	51.5%	43.0%
Family status						
Single, no children	35.0%	38.5%	7.3%	11.1%	2.8%	9.1%
Married, no children	30.5%	33.0%	17.4%	17.5%	59.4%	60.3%
Has Children	34.5%	28.5%	75.3%	71.4%	37.8%	30.6%
Spouse Characteristics*						
Not working	31.9%	6.8%	42.8%	10.1%	43.8%	21.7%
Average Earnings	$49,000	$98,000	$54,000	$151,000	$44,000	$139,000
Professional or Doctoral Degree	37.6%	42.8%	31.0%	46.2%	20.7%	51.0%
Physician, Lawyer or Manager	31.7%	45.5%	25.2%	46.1%	17.4%	46.6%

* Averages of spouse characteristics are for female physicians who were married and had a spouse reporting data

© 2023 Skinner L et al. *JAMA Health Forum.*

eTable 2. Robustness of Earnings Gap Estimates to Alternative Control Variables

	Unadjusted	Adjusted	Adjusted	Adjusted
age 25-34				
Single	8272	8352	8312	7988
	(2448)	(2223)	(2260)	(2149)
Married no kids	7692	2806	3142	6394
	(3078)	(2906)	(2839)	(2785)
Kids	19429	14524	12999	15819
	(3660)	(3535)	(3440)	(3385)
age 35-44				
Single	59271	60506	57684	56558
	(7027)	(7057)	(7066)	(7121)
Married no kids	62012	61870	62426	60994
	(6094)	(5821)	(5914)	(5836)
Kids	91225	88117	87696	86970
	(2463)	(2476)	(2471)	(2451)
age 45-54				
Single	53379	48594	53722	52952
	(9245)	(9294)	(9999)	(9821)
Married no kids	86458	89668	88538	86008
	(5096)	(5168)	(5187)	(5115)
Kids	108627	107981	110492	108322
	(2736)	(2875)	(2915)	(2915)
age 55-64				
Single	31285	31588	36761	37315
	(11046)	(10452)	(12764)	(12827)
Married no kids	86785	87136	91505	88051
	(3606)	(3470)	(3807)	(3767)
Kids	102589	99862	103025	101771
	(4695)	(4807)	(5119)	(5144)
Controls:				
Demographics	no	yes	yes	yes
HRR and rural	no	no	yes	yes
Industry & employer	no	no	no	yes

Notes: Each cell reports estimates of the male-female gap in earnings from a separate linear regression by age group and family status that adjusts for the variables indicated at the bottom of the table. Estimates in the first column are unadjusted. The second column controls for physician demographics (race (9 categories), ethnicity (5 categories), citizenship status (4 categories), spouse's education (6 categories), single year of age and indicators for the year of the survey. The third column adds controls for rural residence (2 categories) and HRR of residence (306 categories). The final column adds controls for industries (3-digit SIC codes) and employer type (incorporated or not incorporated self employed, private for-profit or non-profit, federal/state/local government). Standard Errors reported in parentheses.

© 2023 Skinner L et al. *JAMA Health Forum*.
