# Supplementary material for: Marriage, Children, and Sex-Based Differences in Physician Hours and Income
Source: JAMA Health Forum. 2023 Mar 24;4(3):e230136. doi: 10.1001/jamahealthforum.2023.0136 (PMC10313148; doi:10.1001/jamahealthforum.2023.0136)
Supplement: Supplement 2. — Data Sharing Statement [file jamahealthforum-e230136-s002.pdf]

# Data Sharing Statement

Skinner. Marriage, Children, and Sex-Based Differences in Physician Hours and Income.
*JAMA Health Forum*. Published March 24, 2023. doi:10.1001/jamahealthforum.2023.0136

**Data**
**Data available:** Yes
**Data types:** Deidentified participant data
**How to access data:** https://usa.ipums.org/usa/
**When available:** With publication

**Supporting Documents**
**Document types:** None

**Additional Information**
**Who can access the data:** Anyone requesting the data, it is publicly available.
**Types of analyses:** Any purpose
**Mechanisms of data availability:** No restrictions, investigator support not needed
